# Supplementary figures and images for: Disease resistance gene count increases with rainfall in Silphium integrifolium
Source: Ecol Evol. 2024 Sep 3;14(9):e11143. doi: 10.1002/ece3.11143 (PMC11371658; doi:10.1002/ece3.11143)

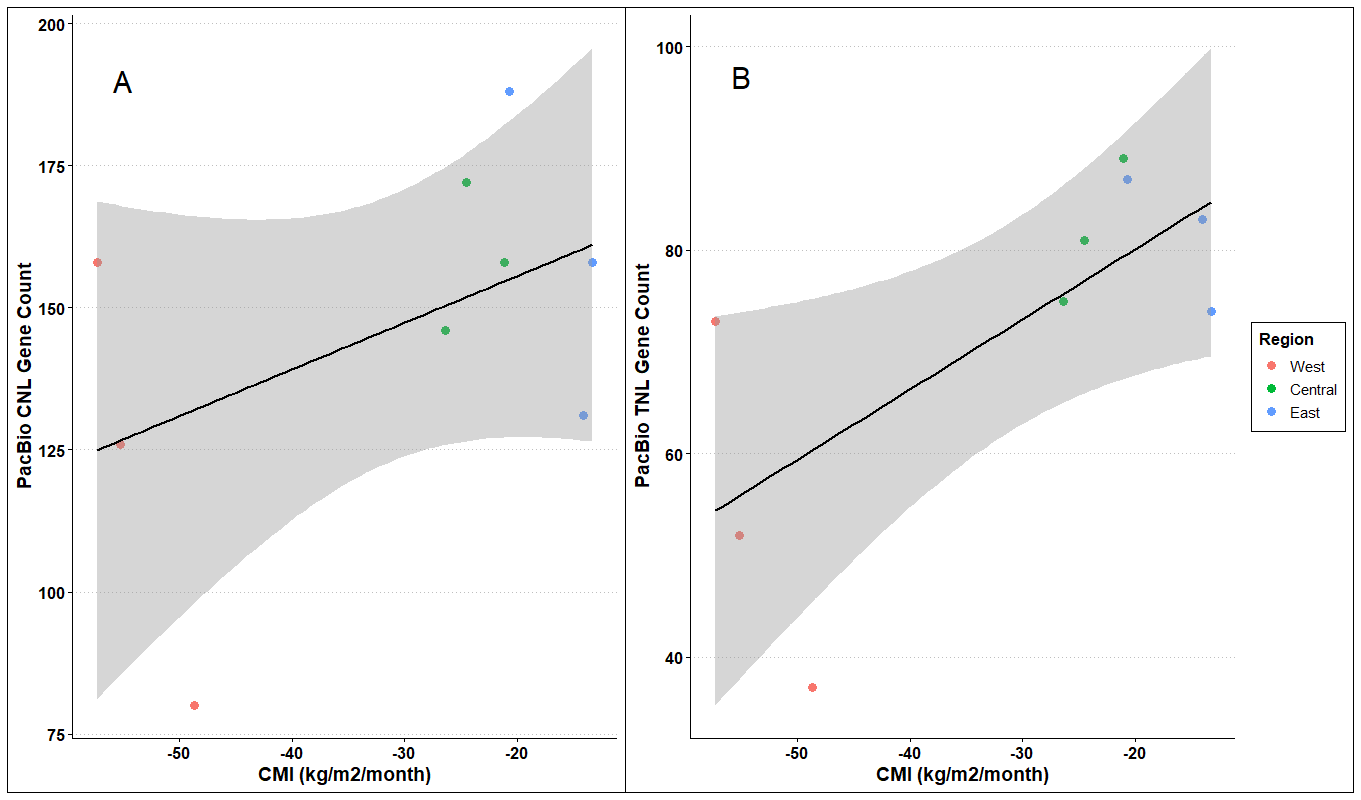

Supplement: Supplementary file 1 — Figure S1 [file ECE3-14-e11143-s002.png]

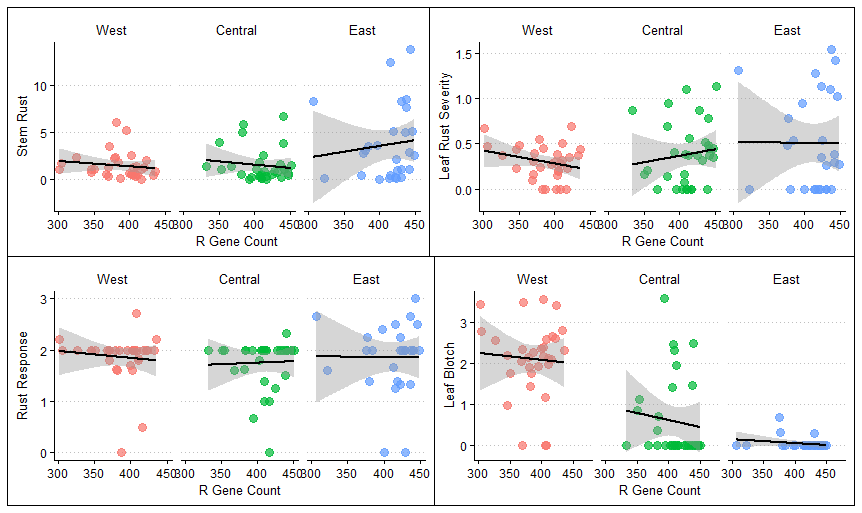

Supplement: Supplementary file 2 — Figure S2 [file ECE3-14-e11143-s007.png]

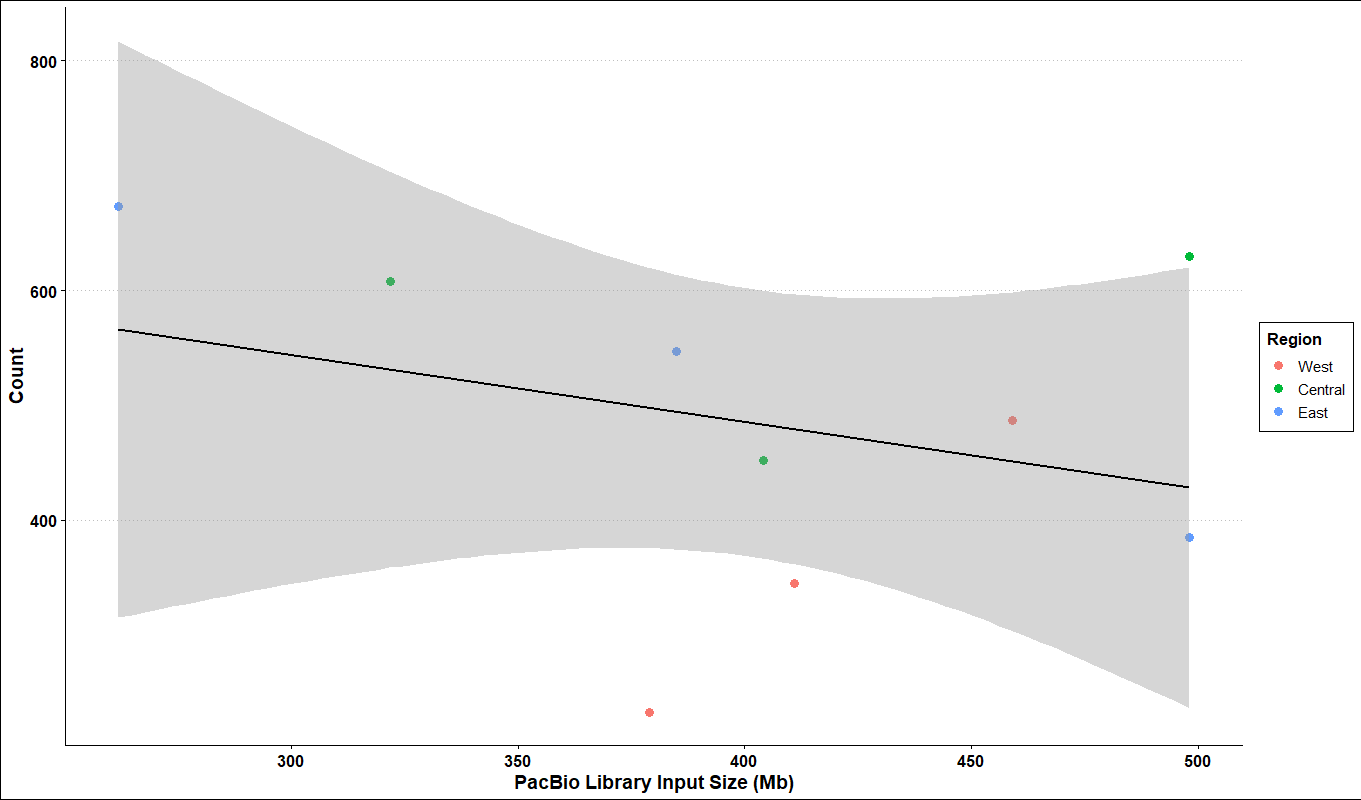

Supplement: Supplementary file 3 — Figure S3 [file ECE3-14-e11143-s008.png]

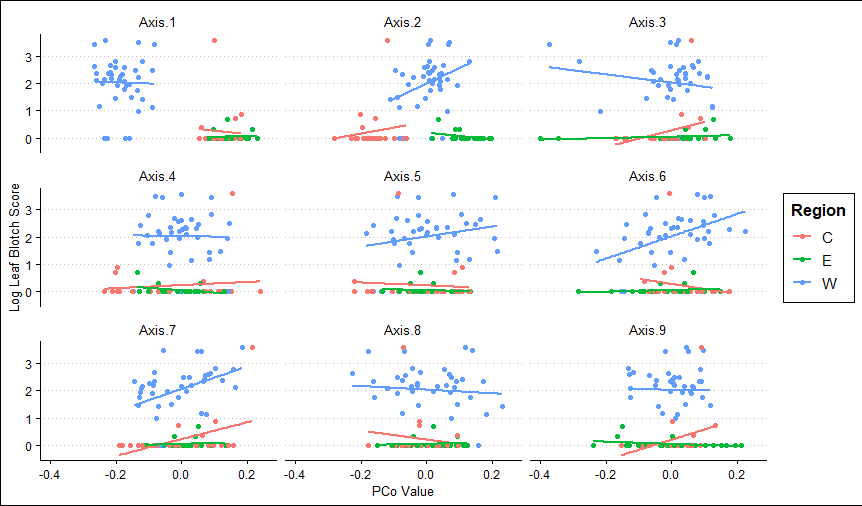

Supplement: Supplementary file 4 — Figure S4 [file ECE3-14-e11143-s006.png]
