# Supplementary material for: Disease resistance gene count increases with rainfall in Silphium integrifolium
Source: Ecol Evol. 2024 Sep 3;14(9):e11143. doi: 10.1002/ece3.11143 (PMC11371658; doi:10.1002/ece3.11143)
Supplement: Supplementary file 8 — Data S1 [file ECE3-14-e11143-s003.zip › Tab s5 caption.docx]

Table S5: Census of the NBARC domains identified in the study. All NBARC domains among the R gene libraries were extracted using NLR-Annotator and each individual was inventoried for the presence (represented as 1 in the table) or absence (as 0) of each NBARC sequence.
