# Supplementary material for: Disease resistance gene count increases with rainfall in Silphium integrifolium
Source: Ecol Evol. 2024 Sep 3;14(9):e11143. doi: 10.1002/ece3.11143 (PMC11371658; doi:10.1002/ece3.11143)
Supplement: Supplementary file 8 [file ECE3-14-e11143-s003.zip › Supplemental Figure Captions.odt]

Figure S1: (A) CNL gene counts and (B) TNL gene counts for the nine PacBio assemblies. In contrast with the Illumina libraries, PacBio TNL counts are significantly associated with CMI (p = 0.018) but CNL counts were not (p = 0.14). This reversal of significance is likely due to the outlying effect of individual W4-07, which contained only 37 TNLs in its assembly.

Figure S2: Disease phenotypes broken down by broad geographic region reveals no dependence of phenotype on R gene count.

Figure S3: PacBio library input size does not correlate with total R gene counts (p=0.414), suggesting there is little need to standardize input size for PacBio libraries beyond a certain input size exceeded by each library in this study.

Figure S4: The first nine principle coordinate axes of the NBARC census data (Table S5) plotted against the leaf blotch scores for Central (C), East (E) and West (W) individuals. No relationships were significant after correcting for multiple tests.

Table S1 (not shown, for size). Master data spreadsheet containing all R gene counts, phenotype scores and CMI that were used to generate the figures and statistics in this manuscript. This table is used as the input for most code blocks in the master R script to replicate these results.

Table S2: Descriptive statistics of R gene counts found within the Illumina and PacBio library types in each of the West (W), Central (C), and East (E) regions. Means are reported with SDs in parentheses. TNL and CNL refer to R genes of type Toll-interleukin-like and coiled-coil-like, respectively. The latter four columns report statistics for how many R genes in each library were classified by NLR-annotator as “complete”, “partial”, “complete but pseudogenized”, and “partial and pseudogenized”. A gene classified as pseudogenized is presumed to be non-functional for containing internal stop codons.

Table S3: Statistics of the simple linear models that were fit to several classes of data. The p-values in these models fail to account for source prairie as a random effect, thus the statistics in Table S4 should be viewed as a more reliable indicator of the effect of CMI on the covariates reported.

Table S4: Mixed effect model statistics. We modeled R gene count (Count), TNL counts (TNLs) and CNL counts (CNLs) as a function of Climate Moisture Index (CMI) as a fixed effect, and source prairie as a random effect (1|source_prairie in our model). The significant correlation of CNL gene counts in our general linear model (p = 0.00073) ceases to be significant when source prairie is modeled as a random effect (p = 0.0627). However, overall R gene count remains significant in our mixed effect model (p = 0.0139). The significant correlation between leaf blotch and CMI in our general linear model (p = 0.0043) remains significant in the mixed effect model (p=3.4e-05).
